# Teratoma with Malignant Transformation: A Case Report with Pathological, Cytogenetic, and Immunohistochemistry Analysis

**DOI:** 10.1155/2011/450743

**Published:** 2011-06-09

**Authors:** Jue Wang, Syed A. Jaffar Kazmi

**Affiliations:** ^1^Department of Internal Medicine, Section of Oncology-Hematology, University of Nebraska Medical Center, Omaha, NE 68198-7680, USA; ^2^Department of Pathology and Microbiology, University of Nebraska Medical Center, Omaha, NE 68198-6495, USA

## Abstract

*Background*. Teratoma with malignant transformation (TMT) is rare and most commonly encountered in adult patient with germ cell tumor (GCT). *Method*. We report a rare case of testicular teratoma with metastatic TMT/embryonal rhabdomyosarcoma (ERMS). A 44-year-old man underwent right orchiectomy which revealed a malignant teratoma, he subsequently had right pneumonectomy with two pulmonary masses containing a high-grade embryonal rhabdomyosarcoma. The patient developed liver metastasis three months after initial diagnosis. He was treated with a chemotherapy regimen with vincristine, dactinomycin, and cyclophosphamide (VAC) alternating with vincristine and irinotecan (VI) with complete resolution of his liver lesion. The tumors were examined with a battery of cytogenetic, immunohistochemical, and molecular assays. *Results*. The malignant cells were immunohistochemically positive for desmin, myogenin, and MyoD1. Molecular cytogenetics of embryonal rhabdomyosarcoma tissue revealed the presence of i(12p). The tumor expressed high level of TOPO2A, TOPO1, MRP1, MGMT, BCRP, ERCC1, RRM1, and TS. *Conclusion*. The activity of topoisomerase inhibitors and the potential usefulness of topoisomerase expression as biomarkers should be further tested in aprospective study.

## 1. Introduction

Teratoma with malignant transformation (TMT) is germ cell tumor (GCT) which underwent malignant transformation of a somatic teratomatous component to histology that is identical to a somatic malignancy (e.g., carcinoma or sarcoma) [1–8]. TMTs are rare and most commonly encountered in adult patients with GCT. The most frequent malignant components associated with testicular GCT are sarcoma [1, 4]. TMTs are usually metastatic at presentation, have a high recurrence rate, and are more aggressive than teratomas without malignant transformation [5–8]. The prognosis is especially poor for mediastinal TMTs and for those with neural or rhabdomyosarcomatous differentiation [1, 2, 7]. Surgical resection is the mainstay of therapy for localized disease, because TMTs are considered to be resistant to radiation and systemic chemotherapy [8–11]. Effective therapeutic strategies targeted to TMT are needed. A case of TMT successfully treated according to a combined modality is presented here along with a description of immunohistochemistry, molecular cytogenetics assays results.

## 2. Case Report

**Figure 1:**
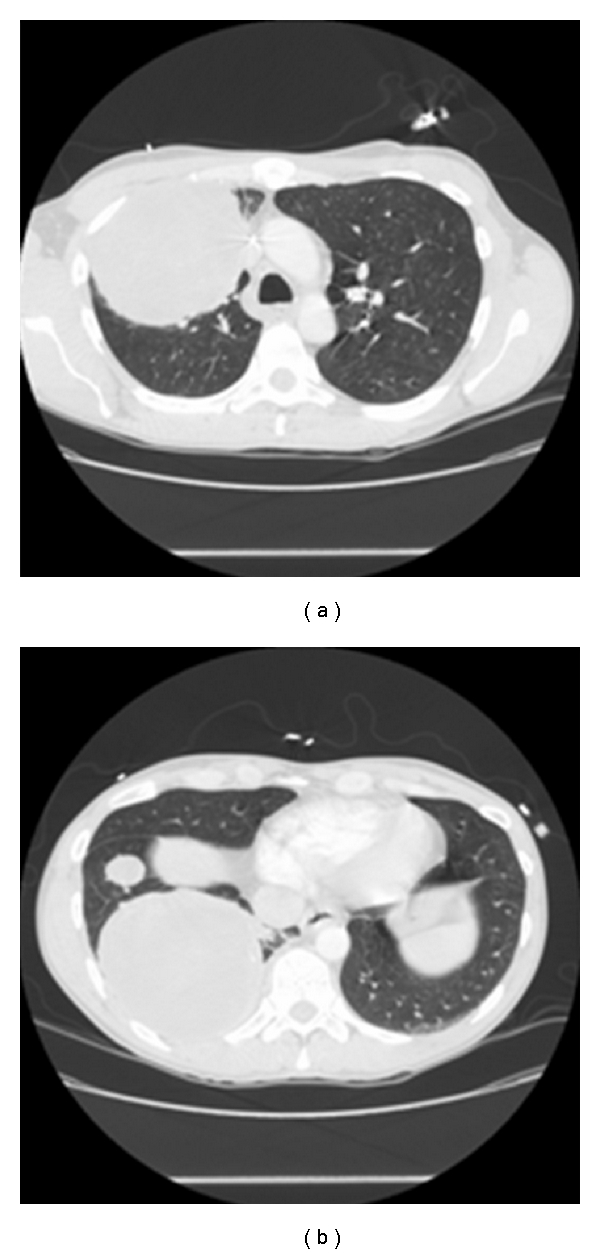
Chest computed tomography (CT) showed large right lung masses; the right upper lobe mass measured 6.8 cm, a large right lower lobe measured 10.5 cm.

**Figure 2:**
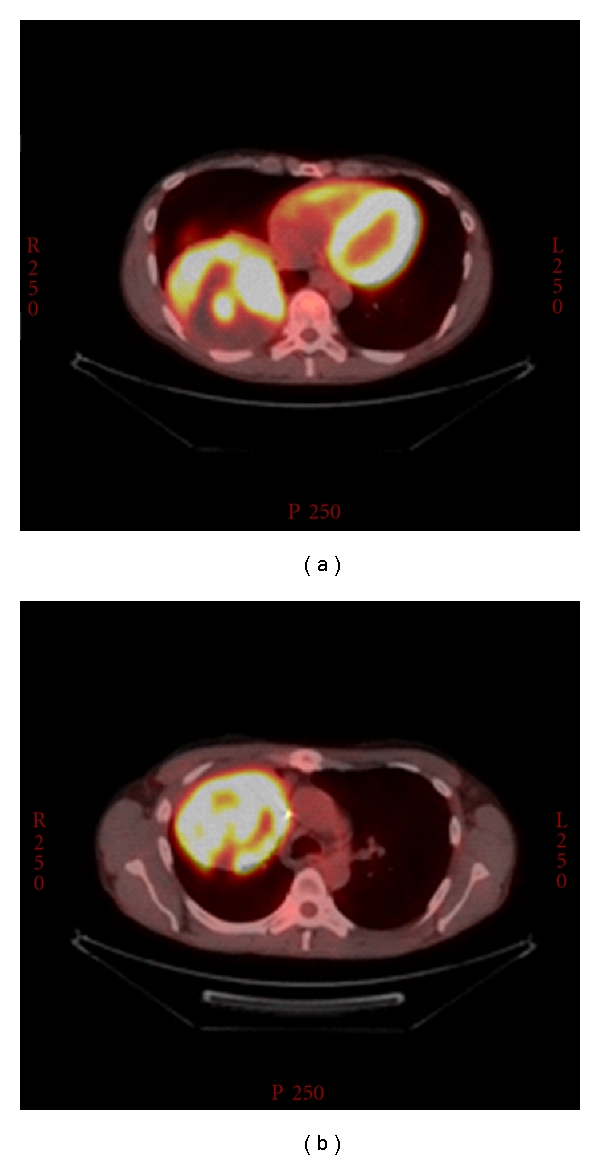
18F-FDG coincidence scintigraphy showed increased FDG uptake in right lung masses.

The patient is a 44-year-old Caucasian male who presented with one-month history of weight loss, cough, pleuritic chest pain and dyspnea. Computed tomography of the chest revealed two right lung masses that measured 6.8 and 10.5 cm (Figure 1). Fine needle aspiration biopsy showed high-grade sarcomatoid malignancy which consistent with embryonal rhabdomyosarcoma. Upon further investigation a right testicular mass was noted. However *α*-fetoprotein, *β*-human chorionic gonadotropin, carcinoembryonic antigen (CEA), and lactate dehydrogenase assays were normal. A right orchiectomy revealed a malignant teratoma. The patient was subsequently transferred to our hospital for chest pain and hemoptysis. A bronchoscopy was performed which did not show any active bleeding, a suspicious endobronchial lesion was biopsied that showed no evidence of malignancy. PET scan showed increase uptake in both pulmonary lesions (Figure 2). The patient subsequently had right pneumonectomy. 

The patient developed a 4 cm liver metastasis two months after pneumonectomy. The patient was subsequently treated according to the arm II of ARST0531 [12] protocol (A Randomized Study of Vincristine, Dactinomycin, and Cyclophosphamide (VAC) versus VAC Alternating with Vincristine and Irinotecan (VI) for Patients with Intermediate-Risk Rhabdomyosarcoma; VAC alternating with vincristine and irinotecan hydrochloride: vincristine IV over 1 minute on day 1 of weeks 1–13, 16, 17, 19, 20, 22–26, 28, 31–34, 37, 38, and 40; dactinomycin IV over 1–5 minutes on day 1 of weeks 1, 13, 22, 28, 34, and 40; cyclophosphamide IV over 1 hour on day 1 of weeks 1, 10, 13, 22, 28, 34, and 40; irinotecan hydrochloride IV over 1 hour on days 1–5 of weeks 4, 7, 16, 19, 25, 31, and 37). He had a complete response of his liver lesion and remains disease-free at 16 months of follow-up after initial diagnosis.

## 3. Pathologic and Cytogenetics Findings

Pathologic examination of the right pneumonectomy specimen revealed two masses measuring 10.1 × 9.8 × 7.4 cm and 10.9 × 9.4 × 8.1 cm, grossly abutting the visceral pleura with a thickened fibrotic capsule. Both of the tumor masses were firm rubbery with homogenous tan-white fibrous fleshy cut surfaces. The tumor obliterated most of the normal lung parenchyma with only scattered residual entrapped alveoli. 

**Figure 3:**
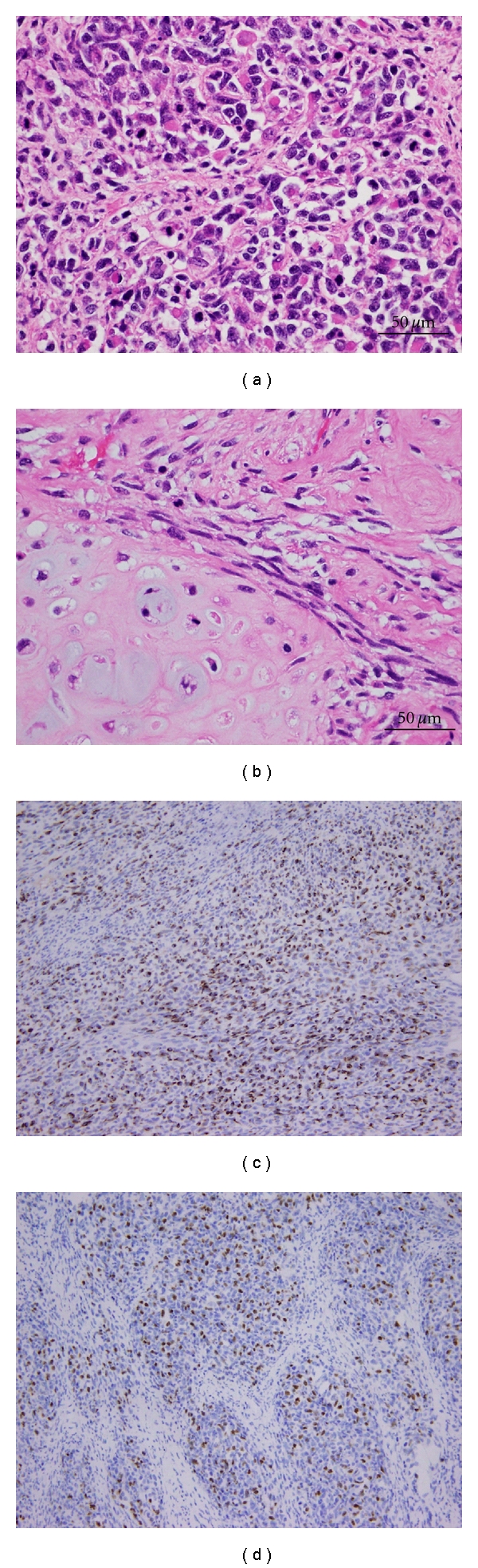
Histopathological findings of the pulmonary tumor. H & E stained section of pulmonary tumor demonstrating rhabdomyosarcoma showing rhabdoid cells (a) and chondrosarcoma with hyaline cartilage differentiation (b). Rhabdomyoblasts are positive for desmin (c) and myogenin (d).

Microscopically, the tumor was composed predominantly of pleomorphic spindle to round cells with scant cytoplasm and round-to-irregular hyperchromatic nuclei (Figure 3(a)). Rhabdoid cells with abundant eosinophilic cytoplasm and eccentric nuclei, were abundant (Figure 3(a)). Rare scattered cells with more terminal differentiation (strap cell) were also noted. Tumor also demonstrated a focal area of chondrosarcomatous differentiation consisting of round to spindle cells with pericellular clearing; foci of hyaline cartilage were also noted (Figure 3(b)). The tumor demonstrated a brisk mitotic activity and multifocal necrosis. Immunohistochemistry showed that the tumor cells were positive for desmin (Figure 3(c)), myogenin (Figure 3(d)), MyoD1, and CD99, and negative for pancytokeratin, CAM5.2, actin, and OSCAR (not shown). The final diagnosis was metastatic embryonal rhabdomyosarcoma with a focal area of chondrosarcoma. Both tumor's masses show the same histology. No GCT was identified. Surgical margins were negative. Multiple lymph nodes were harvested without evidence of malignant involvement. 

Pathologic examination of the testicular mass showed a malignant germ cell tumor composed of teratoma. There was no evidence of immature or somatic type malignant components upon extensive sampling. Fluorescence in situ hybridization (FISH) studies were performed on paraffin sections from lung mass utilizing Homebrew 12p13.2 DNA probe with CEP 12 at the University of Nebraska Medical Center genetics laboratory. Molecular cytogenetic analysis revealed a normal male 46, XY karyotype with presence of i(12p).

## 4. Tumor Profiling by Immunohistochemistry Analysis

**Figure 4:**
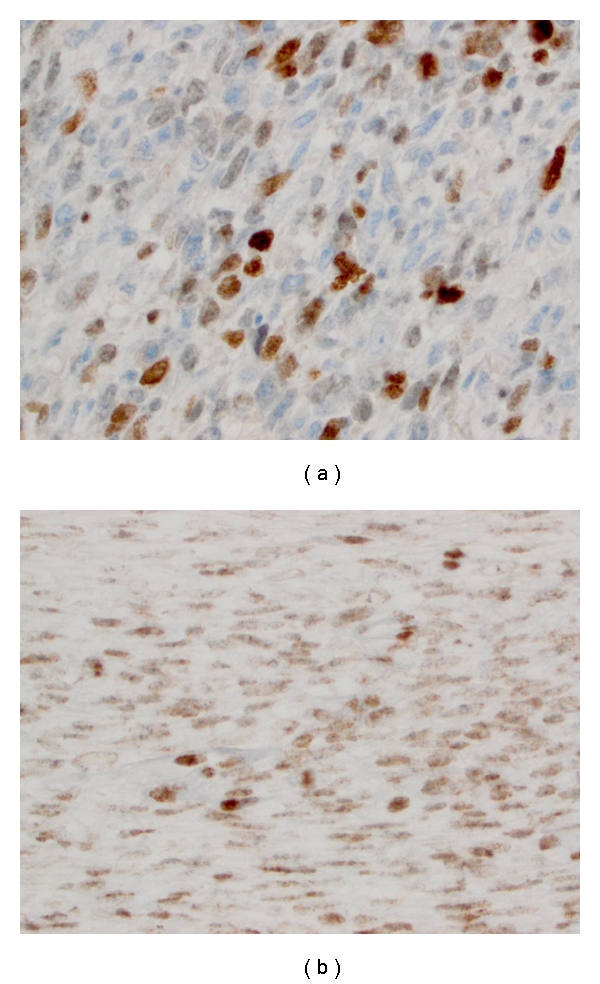
Immunohistochemistry findings of the pulmonary tumor. (a) Rhabdomyoblasts with Topo 2a, (b) Topo I.

In order to identify potential target and biomarkers and therapies associated with clinical benefit, further immunohistochemistry and molecular studies were performed. As demonstrated by Figure 4, Immunohistochemistry study showed high expression of the following proteins: Topoisomerase-II alpha (Topo IIa; 2+, 30%), Topoisomerase I (Topo I; 2+, 10%). In addition, Multidrug resistance-related protein-1 (MRP1) (1+, 60%), O6-methylguanine-DNA methyltransferase, (MGMT; 1+, 90%), breast cancer resistance protein (BCRP; 2+, 90%), excision repair cross-complementation group 1 (ERCC1; 1+, 10%), ribonucleoside-diphosphate reductase large subunit (RRM1; 2+, 80%), and thymidylate synthase (TS; 2+, 50%).

## 5. Discussion

Somatic-type malignancies develop in 3% to 6% of GCT with teratomatous differentiation [1, 4]. It was postulated that they develop from either malignant transformation (MT) of pre-existing teratomatous elements or by differentiation of totipotential germ cells with concomitant MT. The most frequent somatic malignancies associated with GCT are sarcomas, rhabdomyosarcoma being the most common subtype [1, 2]. Up to 80% of patients with TMT present with metastases have a high recurrence rate and poor prognosis whereas others present with unresectable disease [5].

A notable feature of current case is the fact that no sarcomatous component was identified in the testicular mass and no germ cell element was identified in the resected pulmonary tumor. Although extensive histopathologic evaluation was undertaken, sampling error cannot be completely excluded. The presences of two different types of sarcomas (i.e., rhabdomyosarcoma and chondrosarcoma) in the same metastasis favor a GCT origin. The GCT origin of rhabdomyosarcoma in this case is also strongly supported by the presence of i(12p). Chromosomal abnormalities in these TMTs include i(12p), reflecting germ cell tumor clonality, as well as chromosomal abnormalities associated with the transformed histology. Only a few cytogenetic studies have been performed on transformed tumors [13]. Motzer et al. reported i(12p) in 10 of 12 transformed tumors studied including adenocarcinoma, peripheral primitive neuroectodermal tumor, and rhabdomyosarcoma [1]. 

The treatment of TMTs remains a challenge. TMTs have traditionally been thought to be unresponsive to chemotherapy, including cisplatin-containing chemotherapy regimens [10]. Patients who had complete resection of TMTs had significantly better overall survival, and the prognosis for patients with metastatic disease was very poor [3]. Recent reports indicate that chemotherapy directed against transformed component may achieve better results [11]. There have been only a few reports of successful treatment of metastatic TMTs with chemotherapy with long-term survival to doxorubicin-based chemotherapy [10, 11].

Our finding of overexpression of several drug-resistant genes is in line with the clinical observations of lack of response to chemotherapy in these tumors. High expression of MRP1 has been associated with lack of response to etoposide and vincristine [14]. High expression of BCRP has been associated with shorter progression-free (PFS) and overall survival (OS), when treated with platinum-based combination chemotherapy [15, 16]. In addition, genes encode enzyme that catalyzes the biosynthesis of nucleic acid (RRM1), and gene encodes macromolecular target of a cytotoxic drug (MGMT, TS) also over expressed. High expression of RRM1 expression was associated with lack of response to gemcitabine and poor outcome [17]. High expression of MGMT has been linked with resistance to temozolomide-based therapy [18]. High TS has been associated with lack of response to fluoropyrimidine [19]. 

The outcome for patients with metastatic RMS has remained approximately 30% survival for several decades. It has long been recognized that interpatient variability in response to chemotherapy is associated with different outcomes. The complete clinical response with irinotecan-based regimen in this case is interesting. The overexpression of Topo IIa, Topo I in tumor suggested they are attractive targets for novel therapy. Topo I and IIa are enzymes that alter the supercoiling of double-stranded DNA. They act by transiently cutting one or both strands of the DNA. Topo I cuts one strand whereas topoisomerase II cuts both strands of the DNA to relax the coil and extend the DNA molecule [20]. High expression of Topo I has been associated with response to first-line chemotherapy containing irinotecan, a Topo I inhibitor while overexpression of Topo IIa conferred higher probability of response to doxorubicin, a DNA intercalating agent [21, 22]. 

The advent of the human genome sequencing project and the development of high-throughput technologies, including microarrays, have allowed oncologists to explore the possibility of using gene expression profiles to predict chemotherapy drug sensitivity or resistance before treatment, and thereby select the best possible therapies while decreasing the risk of toxicities for the patients. In order to achieve this goal, an international virtual sample bank of this rare tumor is necessary. The Sarcoma Foundation of American Patient Registry and ongoing international efforts to evaluate high-dose chemotherapy in the salvage setting (TIGER protocol) may provide a good opportunity to collect such rare cases. 

In conclusion, we report a rare case of malignant testicular teratoma with metastatic TMT/ERMS. Our findings of overexpression of several drug-resistant genes is consistent with the previous clinical observations of poor chemotherapy response in these tumors. The usefulness of biomarkers for chemotherapy selection should be further tested in a future study.
